# Perforating Granuloma Annulare — An Unusual Subtype of a Common Disease

**DOI:** 10.3390/healthcare2030338

**Published:** 2014-09-04

**Authors:** João Alves, Hugo Barreiros, Elvira Bártolo

**Affiliations:** Department of Dermatology and Venereology, Hospital Garcia de Orta, Av. Torrado da Silva, 2801-951 Almada, Portugal; E-Mails: hbarreiros@gmail.com (H.B.); elvira.bartolo@gmail.com (E.B.)

**Keywords:** granuloma annulare, perforating GA, granuloma

## Abstract

Perforating granuloma annulare (GA) is a rare subset of GA with an unknown etiology and chronic course. Herein, we report the case of 72 year-old women with a 3-month history of a post-traumatic, persistent, erythematous and exudative plaque located on her left leg. Differential diagnosis included mycobacterial infection, subcutaneous mycosis, perforating dermatoses, pyoderma and squamous cell carcinoma. The histopathology was highly suggestive of a perforating GA. The patient was treated with betamethasone dipropionate cream applied once daily and a complete resolution of the lesion was observed in three weeks. Despite being a very rare subtype of a common disease, perforating granuloma annulare has clinical and histopathological characteristic features that facilitate the differential diagnosis, avoiding unnecessary procedures and inadequate and potentially more invasive treatments.

## 1. Introduction

Perforating granuloma annulare (GA) is a rare subset of GA with an unknown etiology and chronic course [1]. It occurs most frequently in childhood and it appears as umbilicated papules that involve, most commonly, the extremities [2]. Treatment could be difficult and unsatisfactory. Due to its rarity, the diagnosis is often difficult and challenging. However, perforating GA has characteristic clinical and histopathological features that facilitate the differential diagnosis, avoiding unnecessary procedures or inadequate treatments.

## 2. Case Report

**Figure 1:**
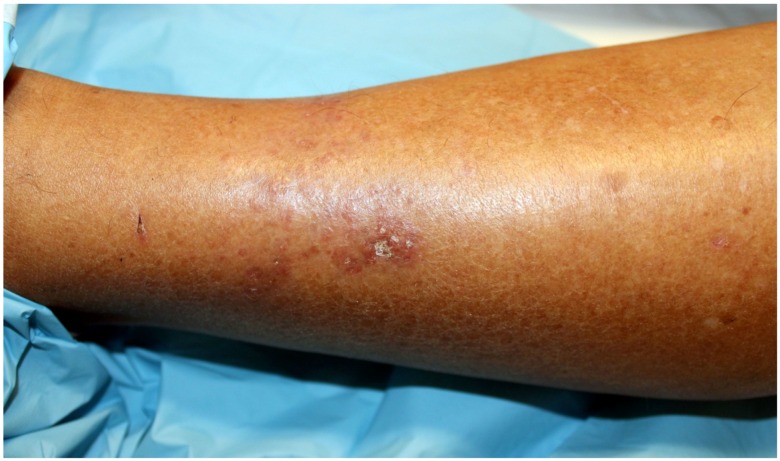
Erythematous plaque centered by erosion covered by crust on left leg.

**Figure 2:**
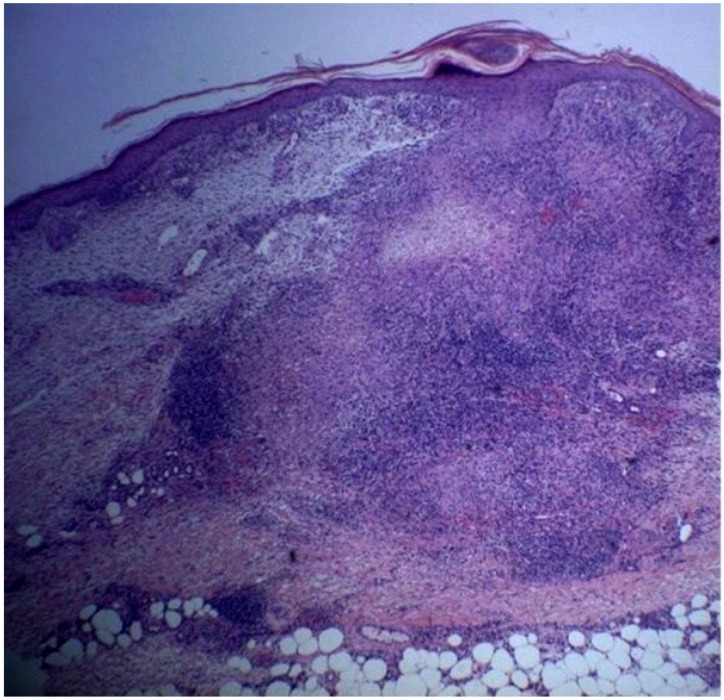
Inflammatory infiltrate composed by lymphocytes and histiocytes occupying the papilar and reticular dermis with palisading granulomas surrounding necrobiotic collagen (H&E 40×).

**Figure 3:**
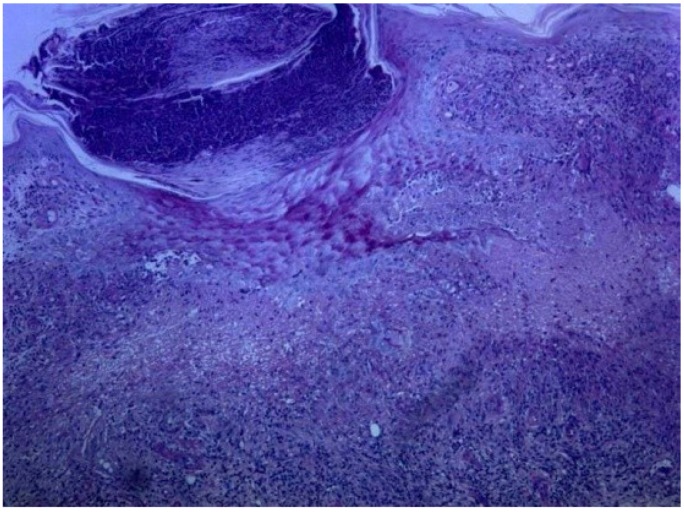
Collagen degeneration and transepidermal elimination (H&E × 100).

**Figure 4:**
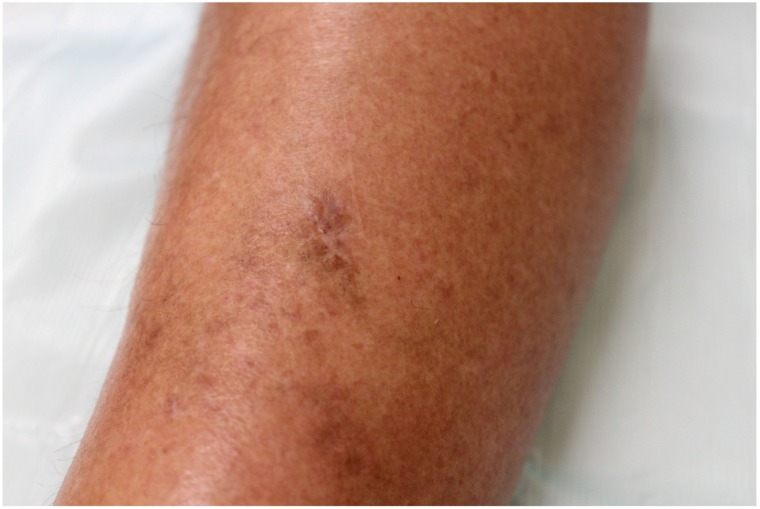
Residual hyperpigmented macule after treatment.

A 72 year-old women was referred to our department due to a 3-month history of a post-traumatic and slightly pruritic erythematous and exudative plaque located on her left leg. She had already been treated with several antibiotics without any clinical improvement. The patient also complained of recurrent gelatinous material extruding from the central area of the plaque. She denied any prior skin conditions or relevant medical history and had no history of recent travel. On physical examination it was observed on the anterolateral lower third of the left leg, a rounded, ill-defined erythematous plaque with 3 cm long axis, centered by small erosion covered by adherent crust (Figure 1). Around the plaque there were some discrete erythematous papules. The differential diagnosis included mycobacterial infection, subcutaneous mycosis, perforating dermatoses, pyoderma and squamous cell carcinoma. A punch biopsy was performed and the histological examination revealed a well-defined nodular infiltrate occupying the papilar and reticular dermis mainly composed of lymphocytes and histiocytes. Collagen degeneration with transepithelial elimination and multiple palisading granulomas surrounding the necrobiotic collagen were prominent (Figure 2 and Figure 3). The exposed features were highly suggestive of perforating granuloma annulare. The patient was treated with betamethasone dipropionate cream applied once daily and a complete resolution of the lesion was observed in three weeks (Figure 4).

## 3. Discussion

**Figure 5:**
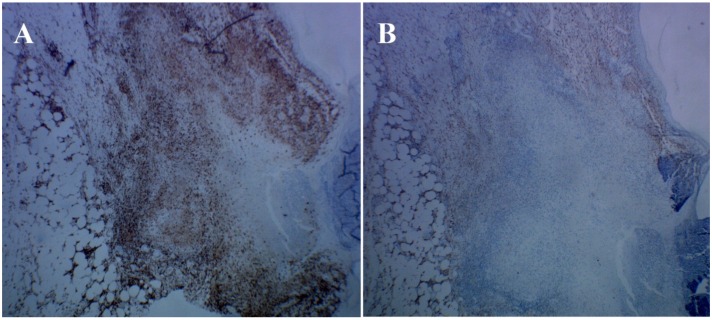
Immunohistochemistry for CD4 and factor XIII-A; (**A**) CD4 cells in the inflammatory infiltrate (immunohistochemistry for CD4); (**B**) necrobiotic collagen surrounded by factor XIII-A+ cells (immunohistochemistry for factor XIII-A).

Perforating granuloma annulare was first described by Owens and Freeman in 1971. It is a rare subset of GA with a chronic course and unknown etiology. It has been suggested that a delayed hypersensivity, helper T cell response to exogenous antigens is responsible for the development of the condition [1]. In our case, T lymphocytes were identified in the inflammatory infiltrate, especially CD4+ cells, which may support this hypothesis (Figure 5A). Some authors suggest that factors such insect bites, ultraviolet radiation, minor trauma, viral infection, thyroiditis and diabetes mellitus are implicated in its pathogenesis [2,3]. Extracellular matrix remodeling is also a key feature in the pathogenesis of GA with large accumulation of macrophages [4]. Factor III-A (FXIII-A)+ CD163+ cells (macrophages) and CD11c+ CD1c+ cells (dendritic cells) are dermal populations cells in the normal human dermis [5]. While FXIII-A+ CD163+ cells were unable to stimulate T cells, CD11c+ CD1c+ cells are typical antigen presenters and are classified as dendritic cells. In samples of GA, FXIII-A+ cells are much more abundant, and the density of CD11c+ dendritic cells are also elevated. Recently, it has been shown that the necrotic areas of GA are mainly composed by CD11c+ cells, which are surrounded by FXIII-A+ macrophages [4]. The high number of CD11c+ cells found in the lesions of GA indicates that, in addition to macrophages, dendritic cells should be taken into account in its etiopathogenesis [4]. A similar finding (necrobiotic collagen surrounded by FXIIIA+ cells) was also found in our case (Figure 5B), suggesting that FXIIIA+ macrophages and, possibly, dendritic cells also play a role in the pathogenesis of perforating GA.

Perforating GA occurs most frequently in childhood and it appears as umbilicated papules that involve, most commonly, the extremities. Sometimes, progressive stages of the disease can be seen: erythematous papules evolve to yellowish pustular lesions which subsequently discharge a clear fluid. A generalized form characterized by larger plaques with necrobiotic centers, ulceration and crusting could be seen in middle-aged and elderly patients and may be associated with diabetes [6]. The clinical differential diagnosis includes molluscum contagiosum, perforating collagenosis, elastosis perforans serpiginosa, perforating folliculitis and sarcoidosis [2]. The histopathological features of perforating GA includes the presence of granulomas with histiocytes sparsely arranged or organized in a palisading pattern, surrounding necrobiotic collagen, which is typically represented by deposition of mucin, and rarely fibrin. Often, the histiocytes may become epithelioid and multinucleate and be found phagocytizing elastic fibers. The process is preferentially located in the superficial reticular dermis, causing perforation of the epidermis which is acanthotic and forming a channel for extrusion of degenerated collagen. Special stains such as alcian blue and colloidal iron can be performed to highlight mucin [3]. The mechanism that leads to perforation remains unknown [6]. Some authors support that the perforation is a consequence of a transepithelial elimination process. Others argue that could represent an epidermal destruction by the superficial GA. Another hypothesis suggests that the superficial localization of the necrobiotic granuloma and the vascular destruction provoked by the granuloma could lead to isquemic epidermal alterations that conclude with epidermal destruction [6]. The histopathological differential diagnosis include granulomatous diseases such sarcoidosis or tuberculids as well as perforating dermatoses [3,6]. The presence of collagen degeneration and multiple palisading granulomas surrounding the necrobiotic collagen without the typical “sarcoidal” granulomas are features more in favor of perforating GA than sarcoidosis [2]. It is important to note that transepithelial elimination could also be present in the latter [7]. The clinical and histopathological features of our case are not suggestive of papulonecrotic tuberculids. Generally, the lesions of papulonecrotic tuberculids are multiple and arise in symmetric crops, typically with acral predilection [8]. Moreover, in histopathology, the vascular damage is much more intense in papulonecrotic tuberculids than in perforating GA [6,8]. Regarding the transepithelial elimination, it could be present in primary perforating conditions (reactive perforating collagenosis and elastosis perforans serpiginosa), in acquired perforating dermatoses which includes perforating folliculitis, Kyrle disease and acquired perforating collagenosis and in other dermatologic conditions that exhibit transepithelial elimination as an incident histopathologic finding (as in GA, necrobiosis lipoidica, rheumatoid nodule and others) [7]. Our case was not in favor of a perforating collagenosis that generally presents as multiple keratotic papules on the extensor surface of the limbs [9]. Histologically, established lesions of this perforating dermatosis, shows a crateriform depression of the epidermis associated with a keratin plug containing inflammatory debris and collagen fibers. Vertically orientated basophilic collagen fibers are seen in the underlying dermis. Palisading granulomas are not a typical feature [9,10]. The clinical (hyperkeratotic papules grouped in an arciform pattern) and the histopathological features (abnormal elastic fibers) of elastosis perforans serpiginosa are different from our case, facilitating the differential diagnosis between them [10,11]. From a clinical point of view, acquired perforating dermatoses such perforating folliculitis, Kyrle disease and acquired perforating collagenosis are less probable because there was no history of an associated systemic disease, as diabetes mellitus, renal failure, liver disease, malignancy or cardiac failure [7]. Moreover, perforating folliculitis is excluded by the clinical (absence of folliculocentric papules with a central keratotic plug in hair-bearing areas) and histopathological features (no involvement of the hair follicle) [10]. The absence of diabetes and renal failure and the presence of the palisading granulomas on the histopathology are also not in favor of a Kyrle disease [10].

Treatment of perforating GA is often difficult and unsatisfactory. The options for localized disease have included high-potency topical steroids or intralesional corticosteroids, although there have not been any studies to determine the efficacy and optimal dosing regimen for these treatments. Triancinolone is probably the most common used intralesional corticosteroid, often at a strength of 5 mg/mL or less [12]. Tacrolimus, pimecrolimus, imiquimod, cryotherapy and simple excision are other reported treatments to localized lesions [1,12,13].

For generalized, severe and recalcitrant forms, other treatments have been proposed with variable success and have included dapsone, clofazimine, niacinamide, potassium iodide, antimalarial drugs, cyclosporine, electrodessication and x-ray therapy [2,12]. Fumarinic acid esters show immunomodulatory activity and affect lymphocytes, dendritic cells and endothelial cell. These effects are thought to be important to the improvement of disseminated GA [14]. Chemophototherapy is another option to treat disseminated GA. Although its mechanism of action is unclear, one possibility is selective elimination of the cells that are responsible for initiating the disease [15]. Due to its proliferative and inhibitory effects on collagen synthesis, retinoids have also been reported as a possible treatment [16]. Methotrexate, regarding its anti-proliferative and, mainly, anti-inflammatory properties, may effectively decrease granulomatous inflammation in GA, and therefore could be another possible choice [17]. More recently, vitamin E and anti-tumor necrosis factor (TNF)-α therapy were pointed as possible therapies to disseminated GA [18,19,20]. Vitamin E modulates inflammatory reactions via reduction of leucocyte recruitment, modulation of nuclear factor kB signaling and decreasing the secretion of interleukin-1b [18]. The efficacy of anti-TNF-α agents is thought to be related to the potentially critical role of TNF-α which is abundantly found in the sera and skin of patients with disseminated GA [20].

## 4. Conclusions

Despite being a very rare subtype of a common disease, perforating granuloma annulare has clinical and histopathological characteristic features that facilitate the differential diagnosis, avoiding unnecessary procedures and inadequate and potentially more invasive treatments.
